# Insulin-Like Growth Factor Binding Proteins in Kidney Disease

**DOI:** 10.3389/fphar.2021.807119

**Published:** 2021-12-22

**Authors:** Shuqiang Wang, Kun Chi, Di Wu, Quan Hong

**Affiliations:** ^1^ Department of Nephrology, Chinese PLA General Hospital, Chinese PLA Institute of Nephrology, State Key Laboratory of Kidney Diseases, National Clinical Research Center for Kidney Diseases, Beijing Key Laboratory of Kidney Diseases, Beijing, China; ^2^ Department of Nephrology, Peking University Shenzhen Hospital, Shenzhen, China

**Keywords:** IGFBPs, kidney disease, function, mechanism, biomarker

## Abstract

The seven members of the insulin-like growth factor (IGF) binding protein family (IGFBPs) were initially considered to be the regulatory proteins of IGFs in the blood circulation, mainly as the subsequent reserve for bidirectional regulation of IGF function during environmental changes. However, in recent years, IGFBPs has been found to have many functions independent of IGFs. The role of IGFBPs in regulating transcription, inducing cell migration and apoptosis is closely related to the occurrence and development of kidney disease. IGFBP-1, IGFBP-3, IGFBP-4 are closely associated with diabetes and diabetic nephropathy. IGFBP-3, IGFBP-4, IGFBP-5, IGFBP-6 are involved in different kidney disease such as diabetes, FSGS and CKD physiological process as apoptosis proteins, IGFBP-7 has been used in clinical practice as a biomarker for early diagnosis and prognosis of AKI. This review focuses on the differential expression and pathogenesis of IGFBPs in kidney disease.

## Introduction

Insulin-like growth factors (IGFs), including IGF-I and IGF-II, are members of the insulin superfamily of growth promoting peptides, and are one of the most abundant and common growth factor polypeptides. IGFs have seven exclusive high-affinity IGF binding proteins (IGFBPs) *in vivo*. IGFBPs exists in blood circulation, extracellular tissue fluid and intracellular tissues, and they can regulate the half-life of IGF in blood circulation, the distribution of IGF in tissues and its binding to cell receptors (Baxter, 2014). It is precisely because of the presence of these seven IGFBPs that complicates the biological utilization and signal transduction of IGF.

IGFBPs is a class of secreted proteins that is able to interact with many ligands other than IGFs, and most of these interactions are believed to be independent of the IGF-IGFR signaling pathway, so these functions of IGFBPs are independent of IGFs/IGFR (Holbourn et al., 2008). At present, IGFBPs research is mainly focused on the tumor field. Multitudes of preclinical studies have shown that IGFBPs can inhibit the growth of tumors, but some studies believe that IGFBPs also exist as an oncogene. In addition, there is evidence that some IGFBPs may be potential biomarkers that can be used to evaluate tumor prognosis or therapeutic resistance.

In recent years, the role of IGFBPs in kidney disease has been taking serious gradually. Studies showed that the growth binding protein family are associated with the development of kidney, primary renal diseases such as mesangial proliferation of IgA nephropathy (IgAN), secondary kidney disease such as diabetic nephropathy (DN), and chronic kidney disease (CKD). Similar to tumors, researchers are also keen to find early diagnosis and prognostic biomarkers for these various kidney diseases. Currently, IGFBP-7 has been used as an early diagnosis and prognostic marker for acute renal insufficiency (AKI). Other IGFBPs are also identified as biomarkers in different kidney diseases (Table 1). This review will review the basic research of the application of IGFBP family in kidney disease, and summarize the research status of IGFBPs in kidney disease, so as to provide some reference for new research.

**TABLE 1:** IGFBPs expression of kidney disease in serum and urine. More and more studies have been conducted on the expression of IGFBPs in renal diseases, mainly focusing on CKD and AKI. All IGFBPs expressions were elevated in the serum of CKD patients, and IGFBP-7 has been a representative marker of AKI, but studies of IGFBPs on primary renal disease still rare.

Disease	Sample	IGFBPs	References
MCD	Urine	IGFBP-1↑	Worthmann et al. (2010)
IgAN	Serum	IGFBP-1↑	Tokunaga et al. (2010)
FSGS	Urine	IGFBP-1↑,IGFBP-3↑	Worthmann et al. (2010)
DKD	Serum	IGFBP-1↑(T1D and DN)	(Gokulakrishnan et al., 2012; Gu et al., 2014; Al Shawaf et al., 2019)
	—	IGFBP-1↓(T2D)	—
	—	IGFBP-2↑,IGFBP-4↑	—
LN	Serum	IGFBP-2↑,IGFBP-4↑	(Wu et al., 2016b; Ding et al., 2016)
AKI	Serum	IGFBP-2↑,IGFBP-7↑	(Bai et al., 2018; Li et al., 2018)
	Urine	IGFBP-7↑	(Kashani et al., 2013; Bihorac et al., 2014)
CKD	Serum	IGFBP-1↑,IGFBP-2↑,IGFBP-3↑, IGFBP-5↑,IGFBP-4↑,IGFBP-6↑	(Fukuda et al., 1998; Ulinski et al., 2000; Dittmann et al., 2012; Sayanthooran et al., 2017; Mirna et al., 2020; Ravassa et al., 2020)

## The Basic Molecular Biology of the IGFBP Family

According to evolution tracking, the homology of IGFBP family genes expanded in the two basal vertebrate tetraploidization (2R) and recombined in the genome of early prochordate animals (Daza et al., 2011). Based on the strict definition of structure and function, there are six recognized IGFBP family proteins named IGFBP-1∼IGFBP-6. Although there is still debate about whether IGFBP-7 should be classified as IGFBPs or defined as IGFBP-related protein (IGFBP-rPs) due to its weaker affinity for IGF-I and IGF-II compared with IGFBP-1∼IGFBP-6, the name “IGFBP-7” is still widely used in the research.

The precursory IGFBP sequence generally has 240–328 amino acid residues before the cleavage of the signal peptide (Daza et al., 2011). Common post-translational modifications of IGFBP family proteins include n-terminal glycosylation, phosphorylation of serine/threonine, and partial proteolysis. The main domains of IGFBPs mainly include IGF binding domains at the amino terminal, heparin binding domains, insulin binding domains and the acid-labile subunit (ALS) in the central region, as well as RGD integrin-binding domains at the carboxyl terminal and nuclear localization domains (Firth and Baxter, 2002). Therefore, IGFBP family has a wide range of molecular functions. In addition to binding to IGF, IGFBP can also bind to cell membrane receptors, integrin family, and play a role directly in the nucleus. IGFBP-3, IGFBP-5 and IGFBP-6 enter the nucleus by binding to the nuclear transporter importin-β through NLS, the same nuclear localization domain at the c-terminal (Schedlich et al., 2000). There is no c-terminal NLS subunit in IGFBP-2, but a structure domain similar to NLS is in the center of IGFBP-2, which is suspected to mediate the nucleation of IGFBP-2 after the combination of importin-β (Azar et al., 2013). However, there is no research showing that the remaining IGFBP are able to import to the nucleus.

## IGFBP-1

IGFBP-1 is the first IGF-binding protein with the molecular weight of 27.9 KDa. IGFBP-1 is high expressed in the female reproductive system and liver, but is low expressed in the kidney. In kidney, IGFBP-1 is mainly expressed in the glomerulus. IGFBP-1 is associated with body and kidney development. All of the whole weight, kidney weight and nephron of IGFBP-1 transgenic mice reduce slightly (Rajkumar et al., 1995). IGFBP-1 plays an important role in diabetes and diabetic nephropathy. IGFBP-1 is associated closely with obesity and insulin resistance (Maddux et al., 2006; Rajwani et al., 2012; Bernardo et al., 2015; Zaghlool et al., 2021). The diagnosis, treatment and prognosis of type 1 and type 2 diabetes mellitus are very different, and the mechanisms of diabetic kidney disease (DKD) are still unclear, the treatment of DKD is a difficulty in clinic. Type 1 diabetes (T1D) and DN had increased circulating IGFBP-1 level and decreased DNA methylation levels of the IGFBP-1 gene. Whereas, low serum IGFBP-1 levels and increased DNA methylation levels in the IGFBP-1 gene were associated with the risk of type 2 diabetes (T2D) (Gu et al., 2014), implies that IGFBP-1 is involved in different types of diabetes by different mechanisms. Glomerulus IGFBP-1 is reduced in early type 2 DKD and controlled by PI3K–FoxO1 activity in podocytes (Lay et al., 2021), thereby to play a role in DKD. In this way, IGFBP-1 may be a promising candidate for diagnoses and therapeutic development in the field of DM and DKD.

In addition, researchers explored the role of IGFBP-1 in other kidney disease. IGFBP-1 expression is elevated in IgA nephropathy, FSGS, acute kidney injury (AKI) and CKD. And it is correlated with estimated glomerular filtration rate (eGFR), cell proliferation (Gleeson et al., 2001), glomerular sclerosis (Doublier et al., 2000; Worthmann et al., 2010), erythropoietin induced stress state (Yamashita et al., 2016) and interstitial fibrosis (Tokunaga et al., 2010). But these mechanism needs to be tested further.

## IGFBP-2

IGFBP-2 mRNA was high expressed in the liver and pancreas, less expressed in the kidney. In kidney, IGFBP-2 is mainly expressed in the mesangial cells. The molecular weight of IGFBP-2 is about 34.8 KDa. Studies of IGFBP-2 focused in secondary nephritis caused by autoimmune disease. Clinical studies found that serum IGFBP-2 is increased in lupus nephritis (LN), but there is controversy in whether IGFBP-2 is related to renal function. In some studies, serum IGFBP-2 level is correlated positively with serum creatinine and can be used as a marker of to reflect the activity and chronic degree of nephritis (Wu et al., 2016a; Ding et al., 2016). But in another study detected by cytokine antibody array, IGFBP-2 is highly related to the activity of SLE and LN, but no significant association with reduced renal function (Yan et al., 2020). This difference in results may due to different race, sample size and detection methods, thus we need more studies to get verification. In basic studies, the increased expression of IGFBP-2 in the outer cortical glomerulus may be associated with glomerular sclerosis and renal loss in lupus nephritis (Mohammed et al., 2003), and it may inhibit the mesangial proliferation induced by IGF-1 and enhance the extracellular matrix deposition (Wolf et al., 2000).

Except LN, IGFBP-2 in the blood can be used as an early diagnostic marker for AKI, and its sensitivity is higher than creatinine, urea nitrogen and cystatin C (Li et al., 2018), and this might be induced by hypoxia (Minchenko et al., 2015). In studies related to chronic kidney disease, circulating IGFBP-2 increased in CKD patients with different conditions, including experimental uremia, CKD caused by heart failure, and children with CKD (Powell et al., 1996; Tönshoff et al., 1997; Mahesh and Kaskel, 2008; Narayanan et al., 2012; Mirna et al., 2020; Ravassa et al., 2020). What’s more, clinical studies have also tracked the renal function level and plasma IGFBP-2 concentration of more than 400 patients with diabetic nephropathy over an 8-year period, suggesting that IGFBP-2 is a biomarker to predict longitudinal deterioration of renal function in patients with type 2 diabetes (Narayanan et al., 2012).

## IGFBP-3

IGFBP-3 is highly expressed in female reproductive system and less expressed in kidney. The molecular weight of IGFBP-3 is 31.6 KDa. IGFBP-3 is the most important IGFBPs in the blood, combining 75–90% IGF-I in circulation (Oh, 2012). Compared with other IGFBPs, IGFBP-3 is involved in a wider range of kidney diseases.

In primary nephropathy, IGFBP-3 is involved in the development of IgA nephropathy and podocytosis. IgA nephropathy is an important type of mesangial proliferative nephropathy. IGFBP-3 was found to be up-regulated in the kidney of experimental IgA nephropathy (Tokunaga et al., 2010). Further research found that in mesangial cells, the expression and release of IGFBP-3 were regulated by IGF and TGF-β. IGFBP-3, TGF-β and IGF form feedback regulation in the glomerulus locally (Grellier et al., 1996). Micropathological nephropathy (MCD) and focal segmentsclerosing nephritis (FSGS) are podocyte diseases. There are few positive podocyte marker (PDX) cells in MCD and more positive cells in FSGS (Worthmann et al., 2010), suggesting that the glomerular podocyte shedding of FSGS is serious, and apoptosis is the important cause of podocyte shedding. Consistent with the excretion rate of PDX cells, the urine excretion rate and the expression in plasma of IGFBP-3 in active FSGS model mice and FSGS patients were significantly up-regulated (Srivastava et al., 2019), IGFBP-3 can be used as a noninvasive biomarker for diagnosis and prognosis of FSGS. In podocytosis, rodent studies have shown that IGFBP-3 regulate the TGF-β/BMP-7 signaling pathway of podocytes and induce apoptosis of podocytes (Peters et al., 2006), what’s more, TGF-β induce up-regulation of IGFBP-3 mRNA expression in human podocytes (Worthmann et al., 2010). In addition, some tumor studies have confirmed that IGFBP-3 promote cell apoptosis through the P53 pathway induced by TGF-β or TNF-α (Besset et al., 1996; Williams et al., 2000). These studies remind us that there might be a positive feedback pathway between TGF-β and IGFBP-3, suggesting a role for IGFBP-3 as a general mediator of programmed death.

The pro-apoptotic effect of IGFBP-3 is also reflected in diabetic nephropathy. IGFBP-3 play an in-depth role in diabetes via apoptosis. Clinical studies showed that IGFBP-3 is down-regulated in Type 2 Diabetes patients with renal disfunction, and predict future renal decline in people with type 2 diabetes combined with apoA4 and CD5L (Peters et al., 2017; Peters et al., 2019). In terms of mechanism, IGFBP-3 mediates high glucose-induced apoptosis by blocking Akt phosphorylation at threonine 308 (pThr308) in mesangial cell and increasing oxidative stress in proximal tubular epithelial Cells (Vasylyeva et al., 2005; Yoo et al., 2011).

Except these, a study including a large population based of 4,028 men and women aged 20–81 years with adjusting for age, waist circumference and type 2 diabetes mellitus showed that IGFBP-3 increases in the blood circulation of in CKD and is negatively correlated with eGFR (Dittmann et al., 2012), However, whether IGFBP-3 is involved in the pathogenesis of CKD or merely serves as a biomarker to indicate the presence of CKD remains to be further studied.

## IGFBP-4

IGFBP-4 is highly expressed in the female reproductive system and the liver, and a little less in kidney. IGFBP-4 has the molecular weight of 27.9 KDa. Like other IGFBPs, IGFBP-4 plays an important role in diabetes mellitus and diabetic nephropathy. People with DN have a significant increase in their plasma IGFBP-1 and IGFBP-4 (Al Shawaf et al., 2019), more importantly, IGFBP-4 fragments (including N- and C-terminal fragments (NT-IGFBP-4 and CT-IGFBP-4)) are related to cardiovascular mortality in type 1 diabetes patients no matter with or without nephropathy (Hjortebjerg et al., 2015). Cardiovascular events are endpoint of death in the vast majority of patients with DN, in this way, IGFBP-4 is potential to serve as a predictive marker for DN patients without affected by their own kidney disease.

Among other kidney diseases, IGFBP-4 was found high expressed in the serum of CKD patients, and this is correlated with the kidney failure degree, the reduced osteogenesis during osteodystrophia (Van Doorn et al., 2001), and growth retardation in children with CKD (Ulinski et al., 2000). Other studies have found that the serum concentration of IGFBP-4 is closely associated with the chronic index of lupus nephritis and the estimated glomerular filtration rate (eGFR), and can be used as a marker for lupus nephritis (Wu et al., 2016b).

## IGFBP-5

IGFBP-5 is highly expressed in the female reproductive system, and the expression of the kidney is medium. The molecular weight of IGFBP-5 is 30.6 KDa. Previous studies showed that IGFBP-5 is highest expressed in mesangial cells (Matsell et al., 1994), thus most of the early studies are concentrated on mesangial cells. The heparin domain of IGFBP-5 mediate the migration of the mesangial cells by combining cdc42 in the high glucose environment (Abrass et al., 1997; Berfield et al., 2000), and IGFBP-5 increases in the glomerular hypertrophy of early diabetes (Schaeffer et al., 2010). But recent studies of single-cell sequencing and our exploration showed that IGFBP-5 is highly expressed in the renal interstitial, which is the highest in kidney vascular endothelial cell and closely related to CKD (Karaiskos et al., 2018; Park et al., 2018). Studies of IGFBP-5 in renal diseases are rare and non-systematic, however, its role in tumor migration, proliferation (Dong et al., 2020) and tissue fibrosis (Nguyen et al., 2018) suggests that IGFBP-5 might be a potential maker which can not be ignored in kidney disease.

## IGFBP-6

IGFBP-6 is the smallest IGFBP with the molecular weight of 25.3 KDa mRNA and protein expression level of IGFBP-6 is high in the kidney. Interestingly, IGFBP-6 can be generated by cleavage of IGFBP-2 by protease in canine renal tubular epithelial cells (MDCK) (Shalamanova et al., 2001). IGFBP-6 was infrequently studied in the kidney, while mostly in proteomic studies of CKD. The abundance of IGFBP-6 in plasma of adults and children with CKD or ERSD were all significantly up-regulated (Jarkovská et al., 2005; Christensson et al., 2018), Consistent with these, the monitoring of plasma IGFBP-6 before and after kidney transplantation and at the time of rejection showed that IGFBP-6 indicate the status of renal function in patients with chronic renal insufficiency (Fukuda et al., 1998), level of IGFBP-6 increases significantly by 8–25 times with the decrease of renal function (Jehle et al., 2000). Meanwhile, the urine abundance of IGFBP-6 gradually increased with kidney developments (CharltonNorwood et al., 2012; Wang and Li, 2015), and is associated with developmental retardation in children with CKD (Powell et al., 1997), suggesting that it was related to the kidney development process. However, these studies did not refer to the mechanism of IGFBP-6 involvement. But IGFBP-6 has been widely studied in tumor and nervous system as a pro-apoptotic protein (Wang et al., 2017; Qiu et al., 2018). Since cell senescence and apoptosis are also important in development and chronic kidney disease, we speculated that IGFBP-6 might be involved in development and fibrosis by regulating apoptosis of renal cells, but more basic research evidence is needed.

## IGFBP-7

IGFBP-7 is high expressed in liver, kidney, bone and muscle, and the expression level is higher in renal tubules. The molecular weight of IGFBP-7 is 29.1 kda. Whether IGFBP-7 should be classified into the IGFBP family is still controversial currently. Because of the weak binding forces between IGFBP-7 and IGF, some studies suggest that they should be classified as IGFBP related proteins (IGFBP-rps). But IGFBP-7 plays a quite important role in kidney, and the main use of IGFBP-7 is the early predictive and prognostic marker for AKI(Bai et al., 2018; Cho et al., 2019). The diagnostic performance of TIMP-2 and IGFBP-7 as biomarkers of AKI was first described in Sapphire study (Kashani et al., 2013). This observational study including 744 patients across 20 North American and 15 European centers led to subsequent clinical study boom on IGFBP-7 and AKI induced by various causes. A year later, the Topaz study prospectively validated the urinary [TIMP-2]•[IGFBP-7] test’s ability (at the 0.3 cutoff level) to identify critically ill patients at high risk for developing moderate to severe AKI within 12 h with the high sensitivity of 92% [95% confidence interval (CI), 85–98%] (Bihorac et al., 2014). Based on Sapphire and Topaz study, the urine compound of TIMP-2 and IGFBP-7 became the first US Food and Drug Administration (FDA)-approved biomarker for risk assessment of AKI in ICU patients in 2014 (Us Food and Drug Administ, 2014). In terms of pathogenic mechanism, TIMP-2 and IGFBP-7 have been confirmed to participate in cell apoptosis by p53, p21, p27 and ERK1/2 signaling as G1 cell-cycle arrest maker during the early phases of cell injury (Kashani et al., 2013; Wang et al., 2018).

## Summary and Outlook

**FIGURE 1:**
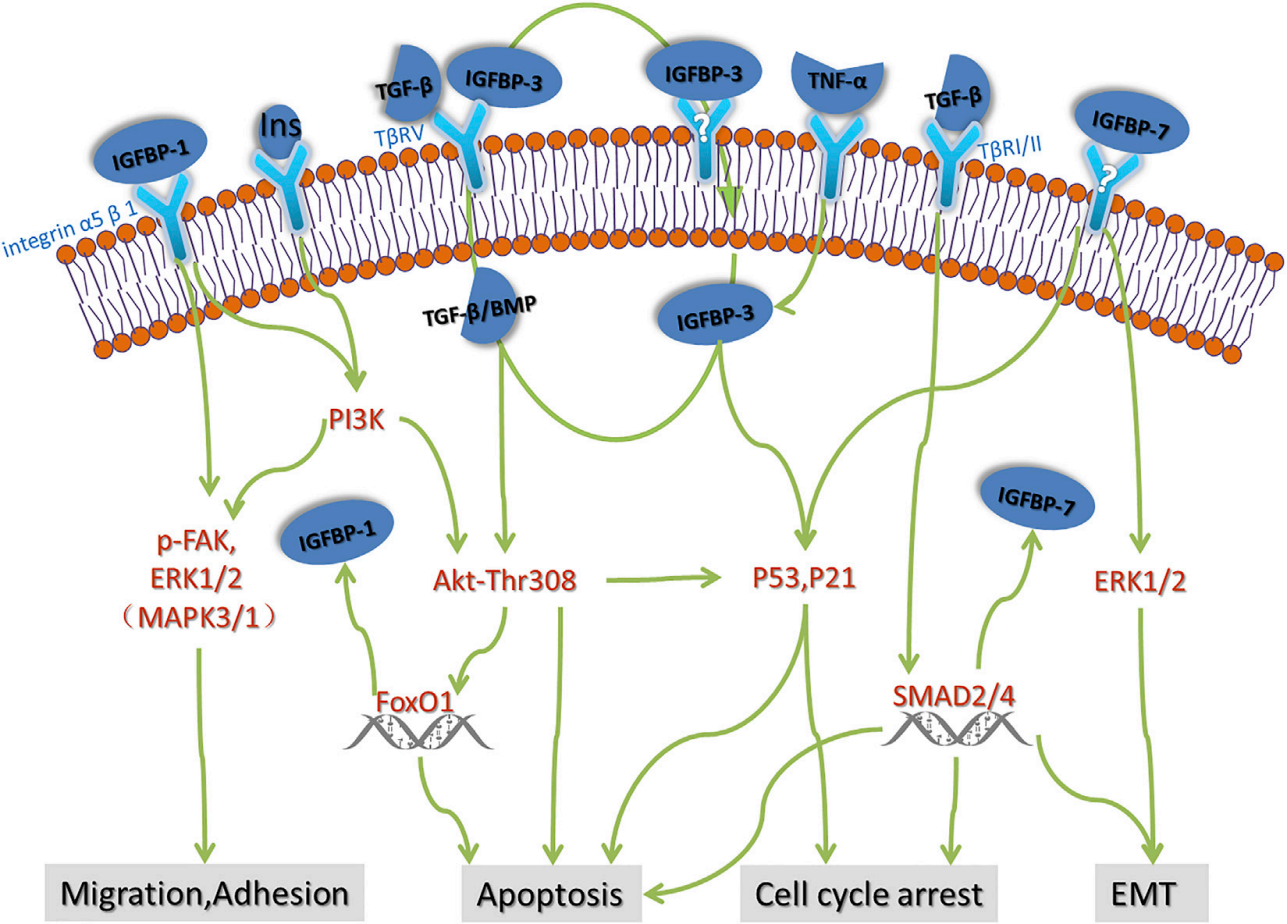
Possible pathogenesis of IGFBPs in renal disease. There are few studies on the mechanism of action of IGFBPs in renal disease. Based on the advances in oncology and rheumatology, we speculate that some of the possible mechanisms of action of IGFBPs in renal disease including cell migration, adhesion, apoptosis, cell cycle arrest and EMT, which need to be further verified by strict basic experiments.

The mRNA expression of IGFBP family is generally low in the kidney, and so far there is less research about IGFBPs in kidney disease, thus the location of most IGFBP in the kidney is not clear. The IGFBP family has a variety of functions, including control the development of the kidney by interacting with IGF, and regulating the biological process of cell proliferation, apoptosis and differentiation independent of IGF, thus participating in the development of IgA nephropathy, podocyte disease, lupus nephritis and diabetic nephropathy. IGFBP-1, IGFBP-3, IGFBP-4 are closely associated with diabetes and diabetic nephropathy. IGFBP-3, IGFBP-4, IGFBP-5, IGFBP-6 are involved in different kidney disease such as diabetes, FSGS and CKD physiological process as apoptosis proteins, IGFBP-7 has been used in clinical practice as a biomarker for early diagnosis and prognosis of AKI. Although the current studies on the mechanism of IGFBPs in kidney disease are still few and unsystematic. We describe the possible pathogenesis of IGFBPs in renal disease in Figure 1 based on current studies. The existing studies suggest that the role of IGFBPs in kidney disease should not be ignored. We believe that future studies will reveal more important functions of IGFBP family.
